# Diagnostic and therapeutic utility of ethiodized oil-based lymphangiography in pelvic and groin lymphatic leaks

**DOI:** 10.1186/s42155-025-00644-w

**Published:** 2026-01-17

**Authors:** Mohammad A. Amarneh, Sara Amro, Kimberly Ferris, Mauricio Amoedo, Ahmad I. Alomari

**Affiliations:** 1 Division of Vascular and Interventional Radiology, Boston Children’s Hospital, Harvard Medical School, 300 Longwood Ave, Boston, MA 02115 USA; 2 Faculty of Medicine, American University of Beirut, Beirut, Lebanon; 3 Division of Vascular and Interventional Radiology, University of Iowa Hospitals and Clinics, Iowa City, IA USA

**Keywords:** Lymphocele, Lymphatic leak, Lymphangiogram, Ethiodized oil

## Abstract

**Background:**

Pelvic and groin lymphoceles and lymphatic leaks remain challenging postsurgical complications. Ethiodized-oil (lipiodol) lymphangiography has been increasingly utilized as a combined diagnostic and therapeutic modality, but published experience with lipiodol-only management in this setting is limited. While transnodal glue embolization is well established, evidence on its long-term outcomes and safety profile remains sparse, with particular concerns regarding the potential risk of lymphedema. These gaps highlight the need for further evaluation of lymphangiography alone as a minimally invasive treatment option.

**Materials and methods:**

This retrospective study included patients who underwent lymphangiography between January 2019 and March 2023 for persistent symptomatic pelvic lymphoceles or groin lymphatic leaks. Imaging findings, drain output, prior interventions, and clinical outcomes were reviewed. Technical success was defined as adequate visualization of the targeted lymphatic vessels. Clinical success was defined as resolution or minimal residual leak without need for further treatment.

**Results:**

Ten patients (5 males, median age, 69 years) underwent lymphangiography for pelvic lymphoceles (*n* = 7) or groin lymphatic leaks (*n* = 3). The median interval from surgery to INL was 67.5 days (range, 12–108). Three patients had previously undergone surgical interventions, and four patients had undergone sclerotherapy without clinical improvement before INL was performed. Technical success was achieved in all patients (100%) with identification of lymphatic leak in all patients. Clinical success was achieved in 7 patients (70%) following lymphangiography alone, with a median time to resolution of 5.5 days (range, 5–12 days) and no immediate adverse events.

**Conclusions:**

Lymphangiography using ethiodized oil contrast is a safe, and potentially effective minimally invasive treatment for pelvic and groin lymphatic leaks. These findings support a stepwise management approach, using lymphangiography as a first-line intervention before escalating to intranodal glue embolization.

## Introduction

Lymphatic leakage from injured lymphatic vessels, presenting as either external drainage (lymphatic fistula) or a lymphocele (contained collection), most commonly occurs after surgical trauma, particularly following extensive lymph node dissection [1, 2]. Most lymphoceles are asymptomatic and resolve without treatment. However, untreated lymphoceles can lead to complications such as pain, swelling, infection, thrombosis, or compression of adjacent structures [3]. The incidence of postsurgical lymphoceles ranges from 1 to 58%, with 5% to 18% of these being symptomatic and requiring intervention [4, 5].

The traditional treatment for symptomatic lymphoceles is percutaneous drainage, with or without sclerotherapy. However, persistent lymphatic leaks often necessitate extended drainage periods. Sclerotherapy, which involves injecting a sclerosing agent such as ethanol or doxycycline through the existing drainage catheter, can be effective, but often requires repeated injection of the sclerosant and prolonged drainage [6–9].

Intranodal lymphangiography (INL) using ethiodized oil contrast is a minimally invasive technique increasingly used for both the diagnosis and treatment of post-surgical lymphatic leaks [6, 10]. Lymphangiography alone can be therapeutic in lymphatic leaks, likely due to inflammatory and embolic effects of lipiodol. However, existing studies often combine embolization or include patients with chylous effusions or ascites, limiting evaluation of an INL-only approach for pelvic and groin lymphatic leaks [6, 11–13]. Our study evaluates a lipiodol-only INL specifically as a first-line strategy for pelvic and groin lymphatic leaks, with escalation reserved for treatment failures.

In this study, we evaluate the effectiveness of lymphangiography with ethiodized oil contrast in diagnosing and treating groin and pelvic lymphoceles, proposing it as a first-line intervention before embolization.

## Materials and methods

This retrospective review was conducted at a single institution from January 2019 to March 2023. This study was approved by the Institutional Review Board (IRB) of the University of Iowa. Informed consent was waived for this study due to its retrospective design.

Data was collected on patients who underwent INL with Lipiodol, an ethiodized poppy seed oil (Lipiodol Ultrafluide, Laboratoire Guerbet, Aulnay-Sous-Bois, France) without transnodal embolization performed during the same procedural setting, for pelvic and groin lymphoceles and lymphatic leaks. Data included demographics, clinical presentation, surgical procedure, lymphatic interventions, length of need for percutaneous drain, drain output prior to and post intervention, and clinical outcome. Imaging studies (ultrasound and CT) performed before and after the intervention were reviewed for all patients with lymphoceles.

### Lymphangiography technique

**Fig. 1:**
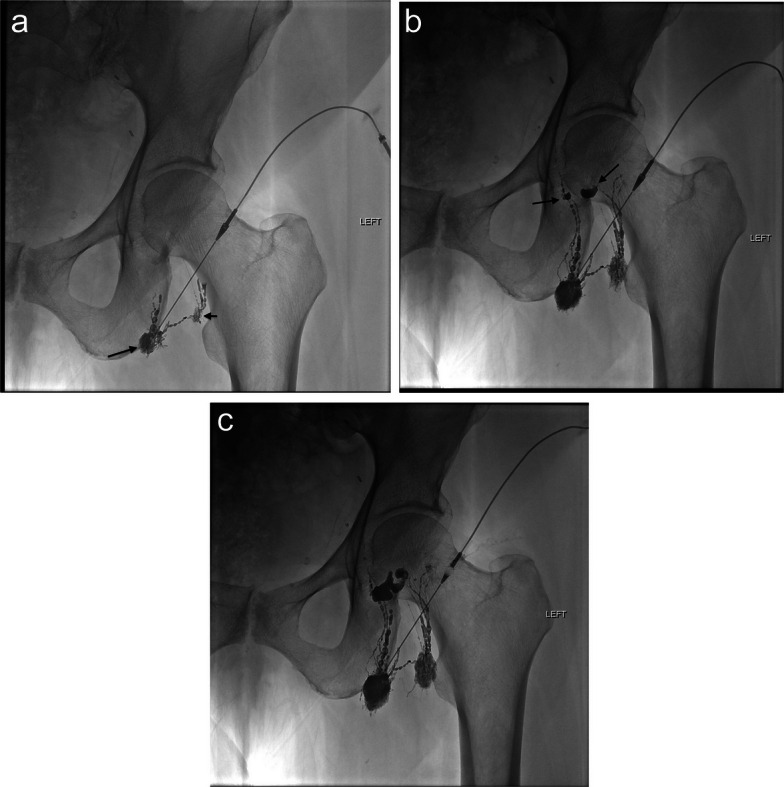
Patient with left groin lymphocele after pelvic lymph node dissection (patient #6). The lymphocele was refractory to percutaneous drainage and 10 sclerotherapy treatments. **a** Early phase demonstrates opacification of the injected lymph node (short arrow), drainage into a lateral lymph node (long arrow), and normal beaded appearance of cranially directed lymphatic channels. **b** Shortly after injection, two areas of lymphatic leakage are seen within the lymphocele (arrows). **c** Later phase shows continued leakage of lipiodol into the lymphocele

**Fig. 2:**
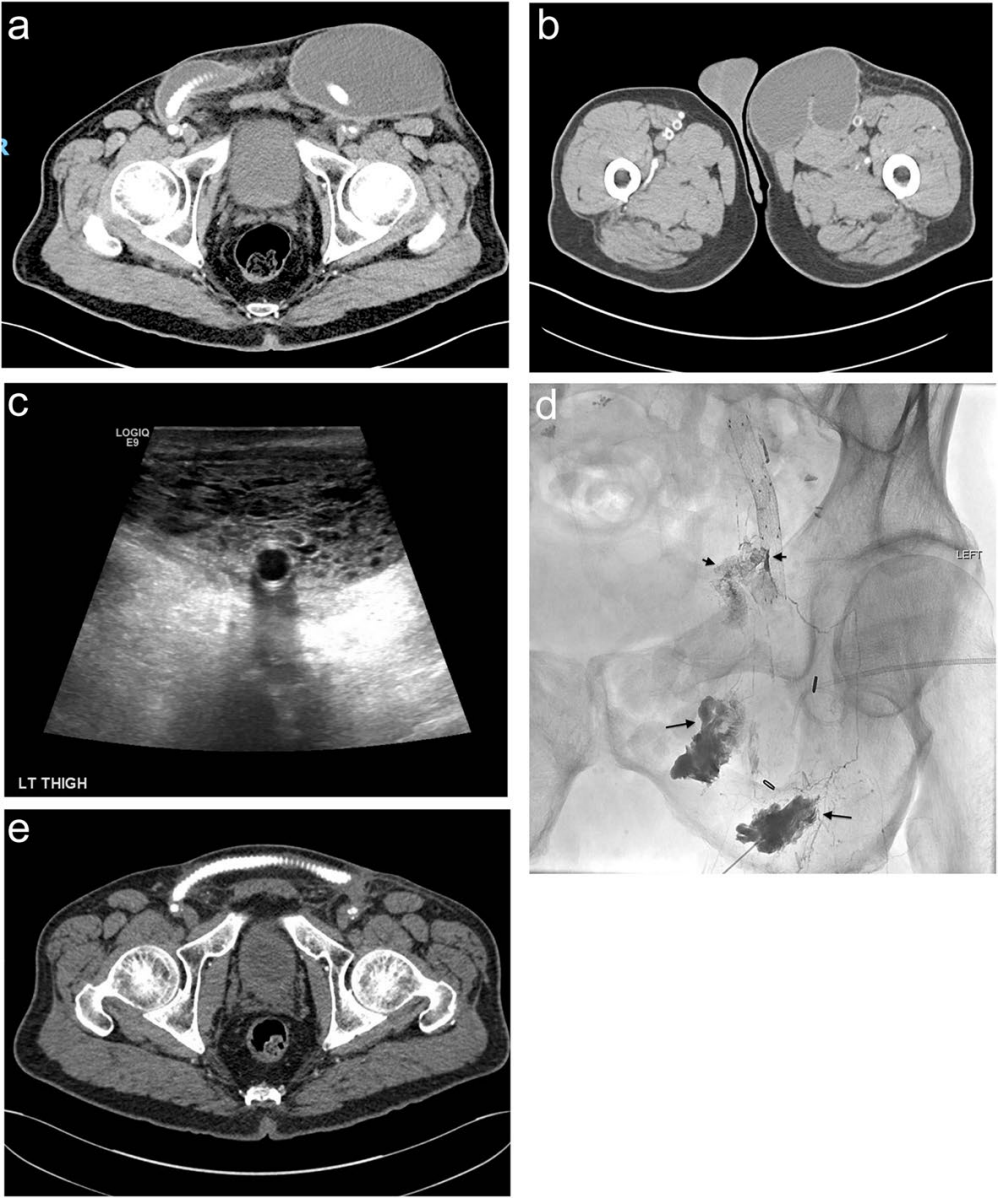
Patient with recurrent left groin lymphocele following femoral artery–femoral artery bypass graft (patient #3). **a** Contrast-enhanced axial CT shows a large fluid collection in the left groin extending around the femoral artery–femoral artery bypass graft. **b** Axial CT at a more inferior level demonstrates caudal extension of the lymphocele. **c** Transverse ultrasound reveals a heavily septated lymphocele within the subcutaneous soft tissues. The femoral artery stent is visible. **d** Lymphangiogram performed via injection into two inguinal lymph nodes shows lipiodol extravasation around the nodes (long arrows) and cranially directed lymphatic channels. There is an abrupt cutoff of a dilated lymphatic channel, with leakage into a spongiform-appearing complex lymphocele medial to the cutoff point (short arrows). A pigtail drain is seen, which was removed at the end of the procedure due to minimal output caused by extensive septations. **e** Follow up CT scan 40 days after the lymphangiogram demonstrates complete resolution of the complex lymphocele

All procedures were performed under moderate sedation and local anesthesia. Under ultrasound-guidance, one or two ipsilateral lymph nodes inferior to the leak were identified and percutaneously accessed with a 22-gauge hypodermic needle (Becton, Dickinson and Company, Franklin Lakes, NJ, USA). Lipiodol was then slowly hand-injected under fluoroscopy at a rate of approximately 0.3–0.5 mL/min until opacification of the efferent lymphatic vessels in the area of interest was demonstrated, with identification of a lymphatic leak when present (Figs. 1 a–c and 2 a–e).

**Fig. 3:**
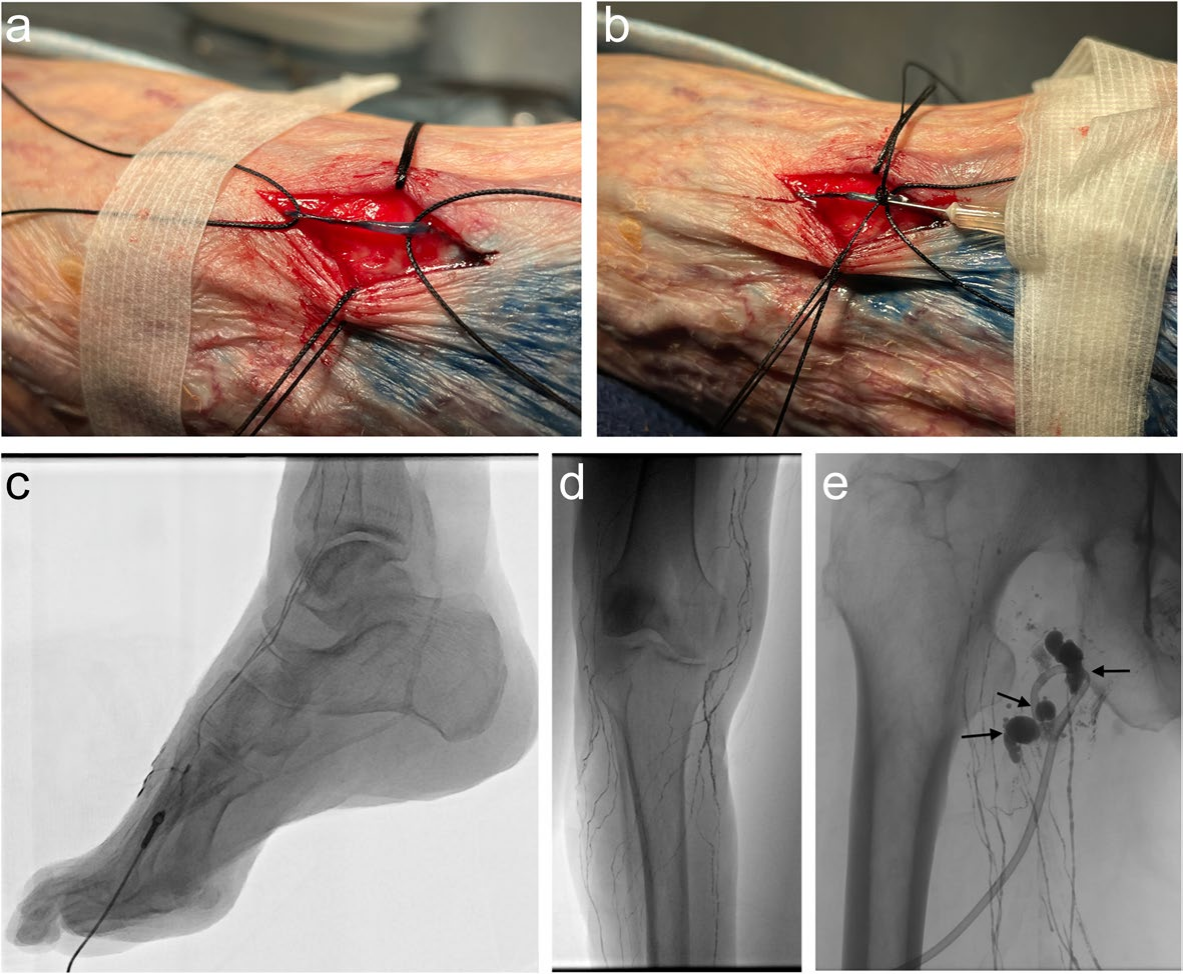
Transpedal lymphangiogram in a patient with a right groin lymphocele following inguinal lymph node dissection (patient #10 in Tables 1 and 2). **a** A total of 3 mL of methylene blue dye was injected into the first and second interdigital spaces of the ipsilateral foot. A lymphatic channel was identified in the dorsum of the foot between the first and second metatarsals. A longitudinal superficial cutaneous incision was made over the channel and exposed using gentle dissection. Two 2–0 silk sutures were placed to retract the incision edges, and two additional 2–0 silk sutures were passed beneath the proximal and distal ends of the exposed channel to lift it for needle access. **b** A 30-gauge needle was inserted directly into the exposed lymphatic channel, and the underlying sutures were tied around the needle to secure access. **c**, **d** Progressive opacification of normal lymphatic channels in the lower extremity with lipiodol. **e** Multiple sites of lymphatic leakage into the lymphocele and surrounding the existing percutaneous drain in the right groin (arrows)

Transpedal lymphangiography was performed in one patient who underwent inguinal lymph node dissection due to lack of visualized inguinal or popliteal lymph nodes. The procedure was performed under moderate sedation. Three milliliter of methylene blue dye was injected in the first and second interdigital spaces of the foot. The visualized lymphatic channel was identified in the dorsum of the distal to mid foot between the first and second metatarsals. A longitudinal superficial incision was made over the channel, which was then exposed with gentle dissection. Two 2–0 silk sutures were used to retract the two edges of the incision. Two additional 2–0 silk suture were placed under the channel’s proximal and distal exposed ends to lift the channel superficially and facilitate needle access (Fig. 2 a–e). A 30-gauge hypodermic needle (Becton, Dickinson and Company, Franklin Lakes, NJ, USA) was directly inserted into the exposed lymphatic channel. The two sutures placed under the lymphatic channel were tied around the access needle. A pediatric infusion pump was then used to inject the lipiodol at 5 mL/hour. A detailed demonstration of this technique is shown in Fig. 3.

Over time, based on early clinical success with lipiodol-only INL, the authors adopted a stepwise approach in which INL with lipiodol was performed as the first-line treatment, with glue embolization reserved for refractory cases.

Technical success was defined as success in visualization of the targeted pelvic or groin lymphatic vessels under fluoroscopy. Clinical success was defined as resolution of the lymphocele or leak, or a negligible residual collection that was asymptomatic and did not require drainage or additional intervention. Immediate adverse events after the procedure were reported according to the Society of Interventional Radiology classification [14].

### Outcome variables

Inpatients were monitored with daily clinical assessment and daily tracking of lymphocele drain output, whereas outpatients were followed with regular clinic or telephone visits and instructed to record daily drain output at home. Decisions regarding repeat imaging, drain removal, or additional intervention were made on a case-by-case basis. Superficial groin lymphoceles were reassessed using ultrasound, while deep pelvic lymphoceles were evaluated with CT.

Descriptive statistics were calculated using Microsoft Excel (Microsoft Corporation, Redmond, WA). For patients with drain output reported as a range, the midpoint of the range was used to approximate daily output. Descriptive statistics including the median, range and interquartile range (IQR) were then calculated based on these midpoint values.

## Results

**Fig. 4:**
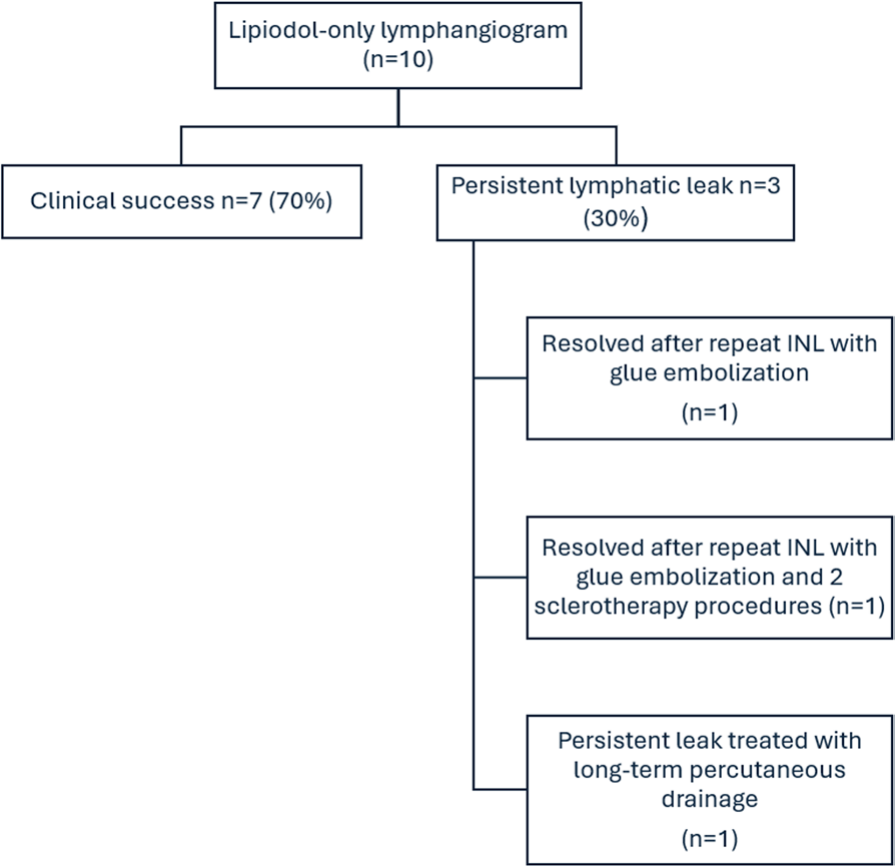
Flowchart summarizing the clinical outcomes

Ten patients (50% men; median age, 69 years; range 27–85 years) underwent 10 lymphangiogram only procedures to evaluate and treat persistent symptomatic post-surgical pelvic lymphoceles (7/10) and groin lymphatic leaks (3/10). Clinical outcomes are summarized in a flowchart (Fig. 4). The demographics and clinical presentation are summarized in Table 1.

**Table 1:** Clinical characteristics of the study population. *CFA* common femoral artery, *PT* popliteal artery, *GSV* great saphenous vein, *ECMO* extracorporeal membrane oxygenation, *DVT* deep vein thrombosis, *TLH* total laparoscopic hysterectomy, *TAH* total abdominal hysterectomy, *BSO* bilateral salpingo-oophorectomy, *HGSC* high-grade serous carcinoma

Patient	Age (years), sex	Lymphocele/lymph leak	Laterality	Diagnosis	Index operation
1	27, Male	Pelvic	Right	Heart transplant, ECMO	ECMO decannulation
2	73, Male	Pelvic	Left	Urothelial carcinoma of the bladder	Open radical cystoprostatectomy, lymph node dissection, ileal conduit formation
2	73, Male	Pelvic, iliofemoral DVT	Right	Urothelial carcinoma of the bladder	Open radical cystectomy, lymph node dissection
3	70, Male	Bilateral groin pelvic, around femoral-to-femoral bypass graft	Left	Peripheral artery disease	Left external iliac artery stenting, right CFA to PT bypass with contralateral GSV, right to left femoral-femoral bypass
4	62, Male	Groin	Right	ECMO, myocardial infarction, other co-morbidities	ECMO decannulation
5	60, Female	Pelvic	Left	Ovarian clear cell carcinoma	TLH BSO, pelvic and paraaortic lymph node dissection, omentectomy, staging biopsies
6	69, Female	Pelvic	Left	HGSC	TAH BSO, rectosigmoid resection, radical tumor debulking, omentectomy, appendectomy, left pelvic and inguinal lymph node dissection
7	85, Female	Pelvic	Left	Urothelial cell carcinoma of the bladder	Cystectomy and lymph node dissection with ileal conduit urinary diversion
8	69, Female	Groin	Right	Idiopathic pulmonary fibrosis s/p lung transplant and ECMO	ECMO decannulation
9	54, Male	Pelvic lymphocele	Bilateral	Penile cancer	Partial penectomy with fasciocutaneous advancement flap and circumcision with bilateral superficial inguinal node dissection
10	79, Female	Groin lymphocele	Right	Vulvar cancer	Vulvar cancer resection with right inguinal lymph node dissection

The technical details, treatment interventions, and outcomes are summarized in Table 2. The median interval between surgical procedure and the INL was 67.5 days (range 12–108 day). Five patients had a pigtail catheter (50%), 2 had a Jackson-Pratt (JP) drain (22.2%), and 2 had a wound vacuum-assisted closure (VAC) in place (22.2%). One patient had a large complex lymphocele around a femoral artery to femoral artery bypass which was not amenable to percutaneous drainage due to extensive septations (Fig. 2a). The median daily drain output prior to the lymphatic intervention was 297.5 mL (Q1–Q3, 125–425; range, 45–1200). Notably, 3 patients had previously undergone surgical interventions, and 4 patients had undergone sclerotherapy with alcohol without clinical improvement before INL was performed. Technical success was achieved in all patients (100%). Lymphangiography demonstrated the lymphatic leak in all 10 patients. The median volume of lipiodol injected was 10 mL (range 1.5 to 13 mL).

**Table 2:** Procedural details and outcomes. Abbreviations: *INL* intranodal lymphangiogram, *Wound Vac* wound vacuum, *NA* not available

Patient	Lymphatic intervention(s)	Prior interventions, days passed since prior intervention)	Additional interventions after INL	Leak on INL	Number of lymph nodes accessed	Total lipiodol volume (mL)	Delay between leak/lymphocele to procedure (days)	Output before lymphatic intervention (mL/24 h)	Output after lymphatic intervention (mL/24 h)	Time from INL to drain removal or leak stop if no drain (days)	Clinical success
1	1. INL	Surgical washout and JP drain placemen, 27	Repeat INL + glue embolization	Yes	2	13	27	115–480	Unchanged	NA	No
	2. Repeat INL lateral LN + glue embolization	INL, 6	None	Yes	1	7	27	115–450	0	2	Yes
2	1. INL	Pigtail drain placement, 8	Repeat INL + attempted glue embolization	Yes	2	6.5	108	300–500	Unchanged	35	No
	2. INL + glue embolization + alcohol sclerotherapy	INL, 6	Alcohol sclerotherapy × 2	Yes	2	6	108	300–500	Less than 300	32	Yes
3*	INL	Surgical debridement for large complex lymphocele, 150	None	Yes	1	7	NA	NA, large complex lymphocele not amenable to percutaneous drainage	NA	NA	Yes
4	INL	Surgical debridement, fasciocutaneous flap to the right groin, followed by rectus femoris myofascial rotational flap and doxycycline sclerotherapy and wound vac, 15	None	Yes	1	8	42	1200	30–40	5	Yes
5	INL	Percutaneous drain placement, 12	None	Yes	1	1.5	38	100	0	12	Yes
6	INL	Pigtail drain placement, 76 alcohol sclerotherapy × 7 and doxycycline sclerotherapy × 3, 15–55	None	Yes	1	4	76	100–200	0	6	Yes
7	INL	Pigtail drain placement, 38	None	Yes	1	5	12	40–50	0	7	Yes
8	INL	Wound Vac, 20	None	Yes	1	5	20	700	0	2	Yes
9	INL	Pigtail drain placement, 36 Alcohol sclerotherapy × 3, (36, 29, 23)	None	Yes	2	10	26	60–100	0	3	Yes
10	Pedal lymphangiogram	Pigtail drain placement, 38 Alcohol sclerotherapy × 3, (38, 29, 23)	Lymphatic channel disruption and glue embolization + ethanol sclerotherapy, followed by ethanol sclerotherapy × 3	Yes	NA	7	108	400–500	Unchanged	NA	No

^*^Patient #3 did not have an external drain due to a complex, septated lymphocele; time to resolution was assessed by follow-up imaging rather than drain output and was not included in drain-based time-to-resolution calculations

Clinical success using INL only was achieved in 7 out of 10 patients (70%). The median time for resolution was 5.5 days (range 5–12). Overall clinical success, including patients who required additional nodal glue embolization or sclerotherapy, was achieved in 9 of 10 patients (90%).

One patient presented with a history of recurrent left groin lymphocele, initially thought to be a seroma and was managed surgically with drainage, irrigation, and debridement. Despite prior interventions, the lymphocele recurred, progressively developing complex septations that rendered the percutaneous drainage ineffective (Fig. 2a–e). INL revealed disruption of lymphatic channels in the left pelvis. Follow-up imaging at 40 days demonstrated complete resolution of the lymphocele, reflecting gradual improvement and resorption rather than a discrete time point of leak cessation (Fig. 2e).

The three patients who had failed the lipiodol-only INL underwent additional interventions to treat the pelvic lymphocele. The first of these patients had a persistent lymphatic leak after the initial INL and subsequently underwent a repeat INL and transnodal glue embolization via the same lymph node, using 25% glue reconstituted with lipiodol. The lymphatic leak resolved 2 days after embolization. The second patient underwent a repeat INL with transnodal glue embolization followed by two sessions of 99% alcohol sclerotherapy before achieving resolution. In the last patient, no lymph inguinal or popliteal nodes were identified which necessitated transpedal lymphangiography with lipiodol, which did not result in clinical improvement. Consequently, two inguinal lymphatic channels inferior to the leak were disrupted using a 21-gauge Echotip needle (Cook Medical, Bloomington, IN, USA) under fluoroscopic guidance, with additional glue embolization performed at the disruption sites, followed by three additional ethanol sclerotherapy procedures without a significant decrease in drain output. The patient was treated with long term percutaneous drainage.

No immediate adverse events related to the procedures were observed.

## Discussion

This study demonstrates that intranodal lymphangiography (INL) with lipiodol alone is a safe and effective treatment for managing persistent pelvic and groin lymphatic leaks and lymphoceles. Of the 10 lipiodol-only INL procedures performed in 10 patients, clinical success was achieved in 7 cases (70%). Two additional patients required repeat lymphangiography with transnodal glue embolization to achieve resolution, while one patient remained refractory despite transpedal lymphangiography, additional lymphatic interventions, and multiple sclerotherapy sessions. No immediate adverse events related to the INL procedures were observed.

Several patients had previously undergone surgical interventions or repeated sclerotherapy without clinical improvement yet responded favorably to lipiodol-only INL. These outcomes support the incorporation of INL earlier in the treatment algorithm for symptomatic lymphoceles. Initially employed primarily as a diagnostic tool, INL became a first-line intervention following a case of rapid and complete lymphocele resolution after a single lipiodol-only procedure in a patient with a lymphocele that did not respond to 10 sclerotherapy sessions over the course of 90 days. Subsequently, a stepwise management approach was adopted: INL was performed first, and glue embolization was reserved for persistent lymphatic leaks. An INL-first approach may be most appropriate in patients with readily identifiable and easily accessible lymph nodes. This consideration is particularly relevant in the post–lymph node dissection setting, where preservation of residual lymphatic function is desirable and immediate transnodal embolization may be deferred.

Reports specifically evaluating INL as a standalone treatment for pelvic lymphoceles are limited, as most published studies include heterogeneous lymphatic leak indications and frequently incorporate adjunctive embolization [13, 15–17]. Verhaeghe et al. reported an 88% overall success rate using lipiodol-only INL in a mixed cohort of patients with pelvic lymphoceles and chylous ascites, with half of pelvic lymphoceles resolving after a single procedure and additional patients responding to repeat INL without complications [15]. Notably, the study employed higher volumes of lipiodol (mean 29.8 mL; range 8–60 mL) and reported no complications. Similarly, Jardinet et al. demonstrated 83% success rates using high-dose lipiodol-only INL for chylothorax, supporting the therapeutic potential of ethiodized oil alone [17]. Campbell et al. evaluated an intranodal lymphangiography-first strategy in a heterogeneous cohort including chylothorax, chylous ascites, lymphoceles, and chyluria [13]. In that study, lymphangiography alone resulted in clinical success in 44% of patients after a median of 14 days, with embolization reserved for refractory cases following a period of observation, supporting a stepwise management paradigm.

In contrast, multiple studies have emphasized early or routine incorporation of transnodal glue embolization following lymphangiography. Moussa et al. reported high clinical success with transnodal glue embolization for pelvic and retroperitoneal lymphoceles, with all embolized patients achieving resolution and drains removed at a median of 6 days [6]. Minor complications including transient fever and ipsilateral lower extremity edema were observed. Similarly, Seyferth et al. reported an 83.3% clinical success rate following transnodal glue embolization, with complications including mild lymphedema and non-target embolization [9]. Hur et al. and Baek et al. likewise reported high overall success using embolization-dominant strategies, though these cohorts were largely composed of chylous ascites and pleural effusions, with embolization performed in most patients once a leak was identified [11, 12]. While glue embolization offers durable lymphatic occlusion, it is associated with a higher risk profile, including permanent lymphatic obstruction and potential non-target embolization. These findings support a selective, stepwise approach in which lipiodol-only intranodal lymphangiography is performed first, with escalation to embolization reserved for persistent or refractory leaks.

Sclerotherapy remains a commonly used treatment for postoperative lymphoceles, though its effectiveness is highly variable. Multiple treatment sessions are often required, and the time to resolution is generally prolonged [7, 8]. In a comparative study, Moussa et al. evaluated 17 patients with 19 lymphoceles treated with sclerotherapy and 29 patients with 30 lymphoceles treated with lymphatic embolization. Clinical success—defined as drain removal after a single procedure—was significantly higher in the embolization group (83% vs. 47%). Additionally, the median time to drain removal was shorter following embolization (6 vs. 13 days). Similarly, Seyferth et al. reported similar findings, with clinical success rates of 83.3% for transnodal glue embolization compared to 65.2% for sclerotherapy [9]. Patients in the embolization group required fewer interventions (mean 1.3 vs. 2.5) and achieved faster resolution (mean 7 vs. 27 days).

Unlike true cysts or lymphatic malformations, lymphoceles lack an endothelial lining [18], potentially reducing the effectiveness of sclerosants that depend on endothelial disruption. In contrast to sclerotherapy, INL targets the leak at its origin by occluding lymphatic inflow, aligning more directly with the pathophysiology of these collections. A case in this series further supports the therapeutic role of INL in achieving lymphocele resolution. Despite prior surgical management and increasing complexity of the lymphocele that precluded percutaneous drainage, complete resolution was achieved following lipiodol lymphangiography alone. This suggests that effectively halting the lymphatic leak may be sufficient to allow gradual reabsorption of lymphatic fluid, even in cases not amenable to further intervention.

Lipiodol’s physicochemical properties contribute to both its diagnostic utility and therapeutic effect. As an oil-based contrast agent, it remains largely confined within the lymphatic system and typically drains into the venous circulation at the thoracic duct, unless diverted by a leak. Although the exact therapeutic mechanism is not fully understood, extravasated lipiodol is thought to induce a localized inflammatory reaction that promotes occlusion of disrupted lymphatic channels. Additionally, it may have a partial embolic effect by reducing lymphatic flow and allowing time for spontaneous leak closure [19].

This study has limitations. It is a single-center retrospective case series with a small patient cohort, limiting generalizability. The heterogeneity of surgical indications and prior interventions introduces potential confounders. However, the interval between prior treatment and lymphangiography was sufficient to mitigate short-term effects in most cases. The absence of a direct comparison group treated with glue embolization limits the ability to evaluate the relative benefits of each approach. Long-term follow-up was not a predefined endpoint, and delayed lymphatic complications, including lymphedema, were not systematically evaluated.

In summary, lipiodol-only INL achieved clinical success in 70% of cases and was well tolerated. These findings support a stepwise treatment strategy that begins with INL alone and escalates to glue embolization only when necessary. Prospective studies are warranted to further define patient selection criteria, optimize procedural parameters, and assess long-term outcomes.

## Data Availability

The datasets generated and/or analyzed during the current study are not publicly available due to patient confidentiality, as all relevant details are included in the tables presented in the manuscript.

## Abbreviations

INL: Intranodal lymphangiogram
CT: Computed tomography
IQR: Interquartile range
IRB: Institutional Review Board
JP: Jackson-Pratt
VAC: Vacuum-assisted closure
